# Retrospective Observational Study Amidst Myriad Conundrums and Myths of Pediatric Headaches: A Critique on Diagnostics and Effectiveness of Interventions

**DOI:** 10.7759/cureus.42424

**Published:** 2023-07-25

**Authors:** Priyal Khurana, Mayank Gupta, Nihit Gupta, Rajiv K Bansal, Vivek Jain

**Affiliations:** 1 Psychology, Christ University, Ghaziabad, IND; 2 Psychiatry and Behavioral Sciences, Southwood Psychiatric Hospital, Pittsburgh, USA; 3 Psychiatry, Dayton Children’s Hospital, Dayton, USA; 4 Pediatrics, Santokba Durlabhji Hospital, Jaipur, IND; 5 Pediatric Neurology, Neo Clinic Children's Hospital, Jaipur, IND

**Keywords:** headache, children, etiology, intervention, outcome, india

## Abstract

Objective

To study the etiological profile of pediatric headaches (PH) in a tertiary child neurology clinic and to determine the utility of diagnostics, interventions, and long-term prognosis.

Methods

Children (ages 4-15) observed over four years were recruited retrospectively. In primary headaches, the headache frequency and impact on quality of life (QOL) parameters at pre-treatment (T1) were compared post-treatment at follow-up (T2).

Results

Of the 311 eligible patients, 285 had primary headaches (Tension-Type Headache {TTH}: 156; Migraine: 129), and 26 had secondary headaches. The mean (±SD) onset age was 10 (±3) years with a male-to-female ratio of 2.3:1. Migraine was more common in children aged less than seven years (17/28) and TTH in older patients (146/283). The most common causes of secondary headache were intracranial hypertension (ICH) in 11/26 patients (four idiopathic intracranial hypertension (IIH), four following aseptic meningitis, three with cortical vein thrombosis), and ophthalmologic causes in 7/26 (of these five had convergence insufficiency). Hypertension was a rare cause of secondary headaches (2/26 patients).

Neuroimaging was performed in 173/311 (56%), primarily for parental anxiety (160/173; 92%), and was abnormal in only four. At T2 (Median time to follow-up: 29 months; Interquartile range: 22-37 months), data were collected in 207/285 patients with primary headaches (TTH: 109; Migraine: 98). In both migraine and TTH groups, there were statistically significant reductions (p-value <0.0001) in headache frequency and QOL parameters.

Conclusion

In our study, TTH was the most common cause of PH. Neuroimaging was normal in most cases. Psychological interventions were effective but underutilized. The symptoms of primary headaches improved significantly over time, despite poor adherence to prophylactic medications.

## Introduction

The global burden of pediatric headaches (PH) in cross-cultural studies has a wide range of prevalence, from 37-57% in younger children to 57-82% in adolescence [1-4]. Recurrent headaches are a common complaint in children and adolescents, with migraines being more prevalent in school-age children and tension-type headaches (TTH) in post-pubertal children [5]. Pediatric headaches are associated with reduced health-related quality of life (HRQOL), including social and academic functioning impairments [6,7]. The HRQOL impairments in children with migraine are akin to those in other chronic illnesses such as cancer [8].

Despite these observations, there is a lack of large hospital-based PH studies from India, and the few dated studies do not provide comprehensive guidance about diagnostics, treatment strategies, and outcomes [9,10]. The current knowledge and scope of clinical practice are based on research from Europe and the United States. These approaches inadvertently lead to marked variance in clinical decision-making due to their limited generalizability [11,12]. 

Most patients in our setup are self-pay walk-ins from both rural and urban areas, mostly belonging to the lower and middle socioeconomic strata, who often ask pointed questions about the cause of primary headaches, the efficacy of treatment, and the long-term prognosis. Besides, there is also an expectation on the part of the families to complete the diagnostic workup and initiate treatment at the first visit itself. Subsequent follow-up visits are rare; therefore, there is often a very narrow window to investigate and intervene.

This study was designed to ascertain the trends in the etiological profiles of PH from a hospital-based pediatric neurology clinic. We also aimed to assess the utility of diagnostic workups, including the controversial use of neuroimaging. A retrospective method was used to assess the utility of diagnostic processes and the efficacy of therapeutics for symptom reduction and overall outcomes. We measured these outcomes using a questionnaire adapted from the validated Headache Under-Response to Treatment (HURT) scale [12].

## Materials and methods

This was a retrospective observational study of all headache patients aged between 4 and 15 years, seen by a pediatric neurologist in an outpatient clinic from October 2015 to October 2019, in a tertiary care hospital.

Patients were subdivided into four headache categories based on the International Classification of Headache Disorders (ICHD) (3rd edition) criteria [13]: 1) migraine, including migraine variants and chronic migraine; 2) tension-type headache (TTH), including chronic and episodic tension-type headaches; 3) trigeminal autonomic cephalalgia and other primary headache disorders; and 4) secondary headaches. The children with primary headaches with overlap symptoms were classified into the group with which they shared the more prominent symptoms (TTH or migraine). Patients with insufficient data in medical records (MR) or seen within six months from the time of the study were excluded.

Design

a) Pre-Treatment Data (T1): The data on headache frequency, need for abortive medications, and impairments were collected from the hospital medical record (MR). The MR has sections with questions (Appendix-Supplementary file) adapted from the HURT scale [12]. This adapted questionnaire was developed after a peer review and piloting process.

It is a standard practice in our unit that all children with headaches are assessed by a pediatric neurologist for a detailed neurological examination including blood pressure. All children (except those with episodic migraine) also consulted an ophthalmologist for an optic disc examination and to rule out hypermetropia and convergence insufficiency (CI).

If the history and neurological examination were not suggestive of a secondary cause, then the families were educated about the lack of utility of neuroimaging. However, when high parental anxiety was observed, Computed Tomography (CT) and Magnetic Resonance Imaging (MRI) were discussed. Informed consent and assent were obtained after discussing the risk and benefit rationale and alternatives with the patient and family.

b) Interventions: Migraine prophylaxis was recommended for children with ≥2 episodes per month. Non-pharmacological interventions (biofeedback or acupuncture) were not routinely recommended. The TTH patients were advised to consult with a child psychologist, and if indicated, they were also referred to a psychiatrist.

c) Post-Treatment Data (T2): A psychologist collected data from families of primary headache patients (TTH and migraine) about the frequency of headaches, response to the treatment, and long-term outcomes. The questions were identical to those at the initial outpatient assessment (Appendix-Supplementary file). This consultation was performed by telephone.

Statistics

The data collected at T1 and T2 were compiled and collated on Microsoft Excel spreadsheets. Descriptive statistics were computed. A Wilcoxon signed rank test (non-parametric test) was used to compare the T1 and T2 data. A two-tailed p-value < 0.05 was considered significant.

Ethics

The institutional review committee (IEC/2021/43) approved the study. Patient anonymity was maintained throughout the study., Verbal consent was obtained from the family prior to the T2 interview.

## Results

**Figure 1:**
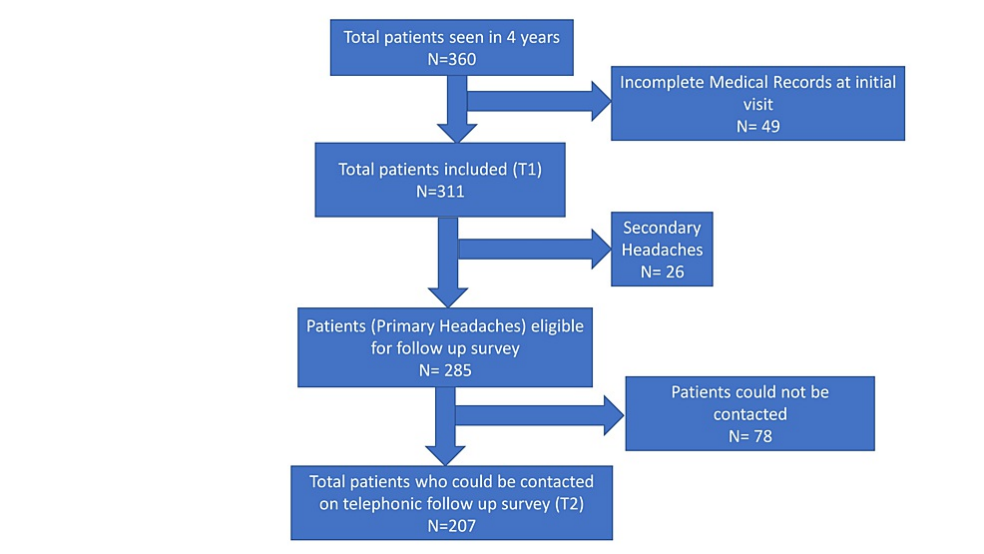
Flowchart of patients recruited

A total of 311 out of 360 patients were analyzed; the rest were excluded due to a lack of information in the MR (Figure 1). The mean (±SD) age of symptom onset was 10.5 (±3) years, with a male-to-female ratio of 2.3:1 (Table 1).

**Table 1:** Patient demographics, clinical characteristics, and yield of Neuroimaging (N = 311) TTH: Tension-Type Headache; SD: Standard Deviation; CT: Computed Tomography; MRI: Magnetic Resonance Imaging; IQR: Interquartile Range; *Abnormal Neuroimaging

	Total (N = 311)	TTH (N = 156)	Migraine (N = 129)	Secondary headaches (N = 26)
Mean Age (years) at presentation (±SD)	10.5(±3)	11 (±3)	10 (±3)	10(±2)
Mean duration (in months) of symptoms (±SD)	13 (±15)	10 (±13)	18 (±7)	2.5 (±2.0)
Mean monthly frequency of headaches (±SD)	15(±11)	23 (±8)	5 (±3)	17 (±5)
Male: Female Ratio	2.3:1	1.8:1	2.9:1	3.3:1
Age distribution	311	156	129	26
4–7 year	28	10	17	1
7–11 year	123	57	53	13
11–15 year	160	89	59	12
Neuroimaging	173	83	74	16
CT Head	163 (4)*	79 (0)	71 (0)	13 (4)*
MRI Brain	37 (4)*	19 (0)	12 (0)	6 (4)*

Of the total 311, 285 (92%) patients had primary headaches, and 26 (8%) had secondary headaches. TTH was the most common headache type [156/311 (50.1%)], followed by migraine (129/311 {41.5%}). Of the 285 patients with primary headaches, 28 also had mixed headaches. Of these 28, 19 were reclassified to TTH and nine to chronic migraine, based on predominant symptomatology. None of the patients had trigeminal autonomic cephalalgia, primary stabbing headache, or any other primary headache subtype.

The three most common causes of secondary headaches (N = 26) were: intracranial hypertension (ICH) in 11 patients, idiopathic intracranial hypertension (IIH) in four, secondary ICH in seven (following viral aseptic meningitis in four, cortical venous thrombosis in three); ophthalmologic causes in seven (CI in five and hypermetropias in two); paranasal sinusitis in five patients. Among the rest, systemic hypertension was documented in two patients. One child had a central nervous system (CNS) malignancy.

**Figure 2:**
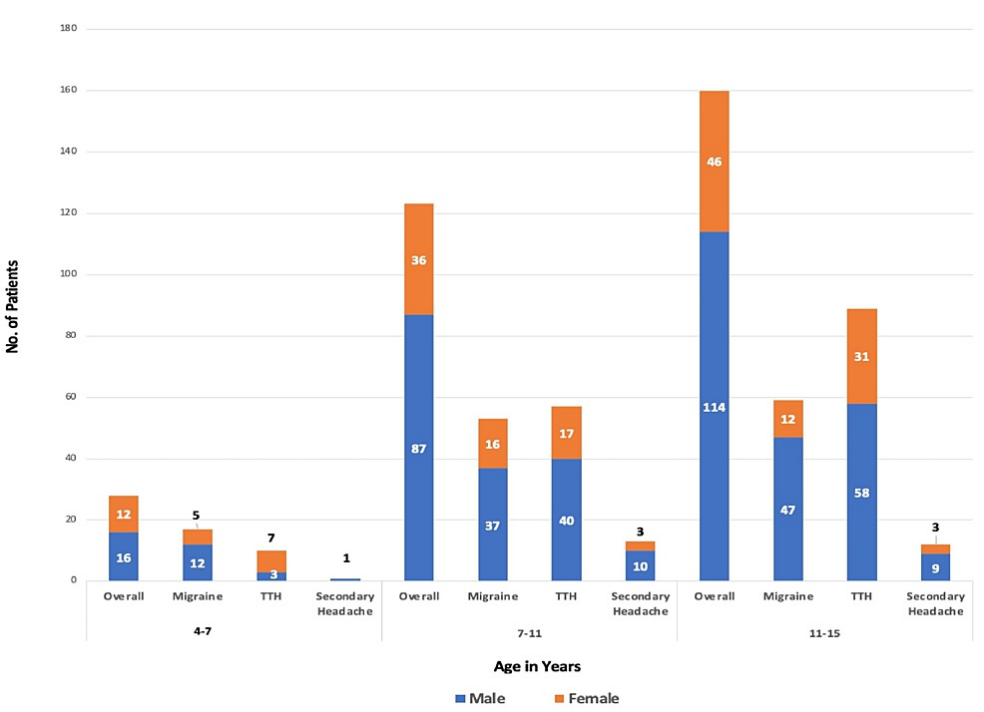
The headache subtypes and gender ratio in three age ranges (N = 311)

The patients were further analyzed in three different age groups (4-7, 7-11, and 11-15 years). When comparing across ages, headaches were more common in children over seven (Figure 2). While migraine was the most common cause of headaches in children under seven (17/28; 61%), TTH was more common (Figure 2) in the older age groups (146/283; 51%).

Neuroimaging was performed in 173 (56%) patients (Table 1), most commonly for parental anxiety (160/173; 92%). In the remaining 13, it was requested to rule out a secondary cause. Neuroimaging was abnormal only in four. These four patients initially had a CT scan of the head and an MRI scan of the brain (three had cortical vein thrombosis, and one had a CNS malignancy). All children with ICH had a normal MRI.

Follow-up data (T2) via telephone contact (Figure 1) could be collected from 207 out of 285 primary headache patients (Migraine: 98/129; TTH: 109/156). In 78 patients, either the contact information was incorrect, or the families declined to provide the information. The 207 patients with both T1 and T2 data available were further compared.

Migraine group (N = 98)

The pre-treatment (T1) median headache duration was 12 months (IQR: 4.5-24 months), with a median headache frequency of two times per month (IQR: 0-4 times) in the previous three months. The median impairment in both daily and social functioning in the preceding three months was four days (IQR: 2-8 days).

Prophylaxis was recommended for 67 (68%) patients. Of these, 63 adhered to the recommended prophylaxis. Flunarizine was recommended for prophylaxis in 40 out of 67 (60%) patients and topiramate in 27 out of 67 patients. The median duration of prophylaxis was 4.5 months (range: 1-18 months). The majority (60/63) stopped prophylaxis without medical consultation after the headache frequency had significantly reduced. The families of three patients did not provide a reason for stopping prophylaxis. Most families could not recall the time for an optimal response after prophylaxis initiation. None of them were on prophylaxis at T2 (Median time to follow-up contact from the first visit: 28 months; interquartile range (IQR) 20-37 months).

**Figure 3:**
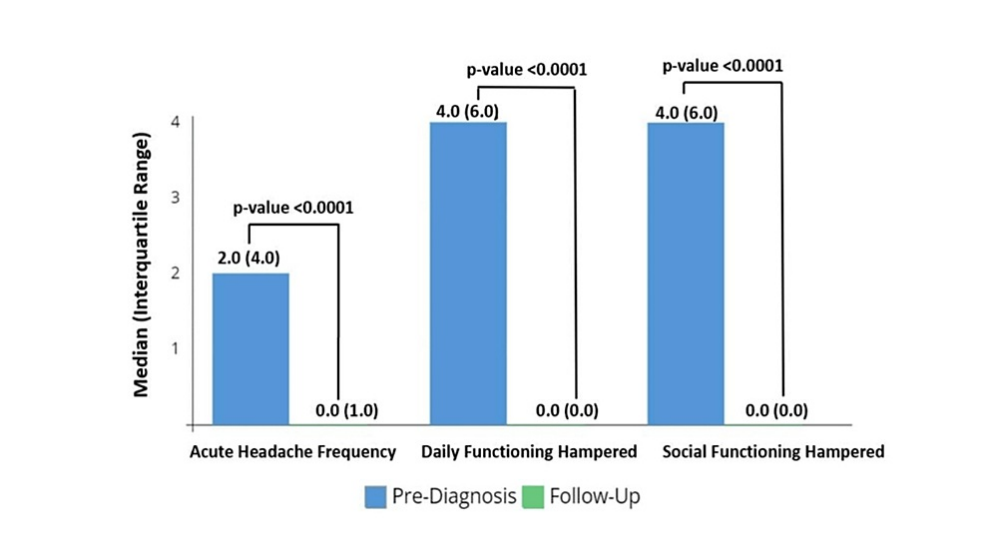
The median change in headache n migraine patients at the follow-up The median change (Interquartile range {IQR}) in headache severity-related parameters in migraine patients (N = 98) at the follow-up telephonic inquiry, as compared to the first clinic visit.

At T2, the median headache frequency had reduced to 0 episodes/month (range: 0-3), a statistically significant reduction (Figure 3; Median difference {T2-T1}: 2 {95% CI 1-3.5}). There were also similar statistically significant improvements (Figure 3; 4.5 {95% CI 3.5-5}) in both daily and social functioning. At T2, 69% (68/98) of families of children with migraine had an insight into the diagnosis.

TTH group (N = 109)

The T1 median headache duration was four months (IQR: 2-12 months), and the median headache frequency was 30 days/month (range: 5-30 days). The median impairment in daily and social functioning in the preceding three months was 15 days (range: 15-30 days).

In total, 94 (86%) patients consulted a psychologist. They had a median of one counseling session (range: 0- 9). Subsequently, the families of 56 patients (60%) continued to use psychological strategies learned during these sessions. 19 patients (17%) used psychotropic medications. The duration of adherence to these medications could not be properly estimated from the parent interviews. None of the patients were on psychotropic medication at T2 (Median: 29 months; IQR: 24-39 months). Most of them could not recall the time taken for an optimal response from T1.

**Figure 4:**
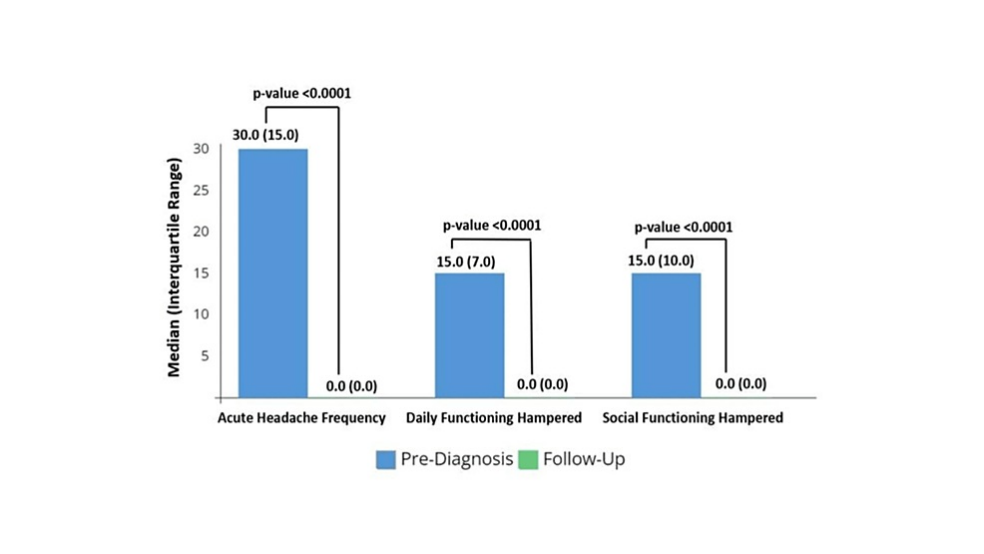
The median change in headache in TTH patients at the follow-up The median change (Interquartile range {IQR}) in headache severity-related parameters in Tension-type headache (TTH) patients (N = 109) at the follow-up telephonic inquiry, as compared to the first clinic visit

The median headache frequency at T2 had reduced to 0/month (range: 0-20/month), a statistically significant reduction (Figure 4; 22.5 {95% CI 22.5-25}). There were also statistically significant improvements in daily functioning (17 {95% CI 15-17.5}) and social functioning (15 {95% CI 12.5-16}) in the preceding three months (Figure 4). Most families with TTH patients (63%; 69/109) also had insight into the diagnosis at the follow-up contact.

## Discussion

Given the paucity of evidence from the subcontinent, there are significant challenges in treating PH. The lack of coherent guidelines has further intensified the debate and confusion among treating pediatricians about evaluation, management, and long-term prognosis.

Pediatric headaches have a multifactorial etiology, which is beyond the scope of this paper, but prior reviews have underscored the presence of marked variability in epidemiology [14]. In our study, TTHs were a more common cause of PH. However, earlier studies on PH (including from India) have reported that migraine is more prevalent than TTH, which adds to the conundrum [10,15,16]. The reason for this could be a missed diagnosis of TTH in many with a classical history of daily headaches by referring pediatricians. In our clinic, the diagnoses for children with migraine were often changed to TTH after initial assessment by the pediatric neurologist.

Likewise, our study showed a significantly higher male prevalence in all age groups and PH subtypes. This indicates a potentially biased situation because male children in India receive better medical attention [15]. In our cohort, the group below age seven had more migraines; the school-agers and teenagers were diagnosed more often with TTH, consistent with previous studies, especially recent similar work from India [16-20]. Only a few of our subjects had secondary headaches, which is also consistent with previously published works [20-21]. However, two subgroups of our patients with secondary headaches have not been well described in the literature.

Firstly, children with a presumptive diagnosis of secondary ICH are associated with aseptic (presumed viral) meningitis. It has been postulated that an inflammatory process, either due to an acute CNS viral infection or secondary to an immune-mediated response, may disrupt the absorptive mechanism of the arachnoid granulations [21]. This may decrease the absorption of cerebrospinal fluid (CSF), resulting in intracranial hypertension (ICH) [21]. These patients with aseptic meningitis and ICH may present with severe acute-onset headaches but are rarely reported, and hence remain underdiagnosed [20-22]. A CSF drainage followed by a short course of acetazolamide may often completely remit the symptoms [21}, as was also the case in all our patients with aseptic meningitis and ICH.

The second group of PH patients which have often been underdiagnosed are the children with convergence insufficiency (CI). In our cohort, it was a more common ophthalmologic cause of PH than hypermetropia. CI has been previously reported as an etiology of PH but is still often overlooked [23,24]. Therefore, testing for CI must be a standard practice. Given that we identified and successfully treated these children (with convergence exercises), we suggest ruling out CI and not just hypermetropia in children with headaches.

Neuroimaging was performed on a large proportion of our patients, often due to parental anxiety, without a clinical rationale and with mostly normal findings. This underscores its limited value in clinical practice, which is also reiterated in the literature [25,26]. These results and the rarity of secondary headaches could be used to educate families, about their anxiety about a significant intracranial cause. This will also help avoid unnecessary investigations in an already burdened healthcare system.

Another frequently asked question in our practice is about the response to interventions in PH and its long-term prognosis. Finding the answer to this was also an important aim of this study.

At T2, most children with TTH had a significant reduction in headache frequency. These findings were counterintuitive, as the patients referred to us had more severe presentations. Although these are encouraging findings for educating the families, we could not rule out the limitations of cross-sectional design and recall bias, as another follow-up study with a smaller sample size reported a poorer long-term prognosis [27,28]. However, these variations could also be attributed to the low power of these studies and possibly cultural and geographical factors. About 90% of TTH patients had at least one session with a trained psychologist as per our recommendations at T1. The poor follow-up rates could be due to stigma, economic stress, or accessibility. But interestingly, 50% of families reported continuing to use strategies learned during consultation. About 17% of patients from this group were prescribed Selective Serotonin Reuptake inhibitors (SSRIs) for co-occurring mental health conditions.

At T2, the migraine group also demonstrated a statistically significant reduction in headache frequency and improvement in associated HRQOL parameters. The current international consensus guidelines are to initiate prophylaxis if the headache frequency is 3-4 times per month for a period of 6-12 months [29,30]. In our clinical practices, there is a deviation, as prophylaxis was started at a lesser headache frequency (≥2 attacks per month). This could be due to the caregivers giving in to higher expectations and lacking local standard hospital guidelines, although emerging evidence recommends prophylaxis for ≥ 2 days of migraine per month [31]. Interestingly, adherence to the prophylactic regimen was < 6 months in our migraine patients, generating a research hypothesis about whether shorter migraine prophylaxis is also as effective or if prophylaxis might have a placebo effect.

Our study, however, had several limitations and weaknesses. There were diagnostic challenges in discriminating between chronic TTH and chronic migraine without aura. These conditions co-occur in many patients with frequent headaches. Based on the predominant symptoms, we classified them into either the TTH group or the migraine group, a potential diagnostic bias. The selection bias was implicit, as all patients were seen at the tertiary center and were predominantly male. The recall bias was due to reliance on memory in collecting T2 data by telephonic interview after a long median follow-up of two years. Also, there needed to be longer-term data about any recurrence of symptoms. The time to follow up for the T2 data was also different for each participant from the initial visit, although within a narrow IQR of 20 to 37 months.

## Conclusions

In clinical settings, contrary to earlier findings from the subcontinent, TTH was the most common type of PH. The variability in prevalence and gender differences of PH underscores multifactorial etiology including sociocultural factors. Amidst many diagnostic challenges and changing nosology, neuroimaging appears to be of little value, while clinical assessment remains the gold standard for diagnostics. With appropriate screening, some individuals may benefit from often underutilized psychological interventions. In our study about 57% of families continued to follow the recommendations of the mental health team which included a mix of cognitive, behavioral, and supportive strategies. These promising findings stress the earlier role of trained mental health providers in treating TTH patients. Lastly, in our study, the symptoms of primary headaches improved significantly over time even after poor adherence to prophylactic medications. 

Despite limitations, this study underscores the role of robust clinical history-taking and cost-effective bedside examination in diagnosing PH. It is also the first sizeable hospital-based follow-up study of PH from the subcontinent amidst challenges in deviant practices, poor follow-up, and intervention variances. It provides the trends of prevalence, subtypes of PH, the effectiveness of interventions, and the long-term prognosis. This study also highlights the effectiveness of the often-ignored psychosocial issues among children and youth in our demographics. These findings inspire many leads for further scientific inquiries into common neurological presentations in children and youths.

## Appendices

Supplementary file

Headache proforma, including questionnaire at initial assessment and follow up

Patient name: Age:

Headache Clinic Questionnaire Date of Clinic........................

Diagnosis: ....................................................................................................

History (from questionnaire responses in the medical records)

Frequency of headaches/month (last three months) ....................................

How many times in the last three months have you taken analgesics for treatment ..............................?

On how many days in the last three months did your headaches make it hard to study or carry out daily activities? ..........................

On how many days in the last three months did headaches spoil or prevent family, social, or leisure activities? ................................

Any Intervention suggested                          Yes                                           No

            (From medical records)

If yes:

Migraine prophylaxis                                     Yes                                           No

           (From medical records)

How long did you take prophylaxis?          ……………………………………………..

           (From telephonic contact)

Psychotropic medication                                 Yes                                        No

           (From medical records)

How long did you take psychotropic medication?........................

           (From telephonic contact)

Psychotherapy                                                   Yes                                        No

(From Telephonic contact)

Pre (T1) and post-intervention follow-up (T2) Questions:

At the initial clinic visit (From medical case records) T1

Frequency of headaches/month (last three months)

Frequency of acute medication intake/Month (last three months)

On how many days in the last three months did your headaches make it hard to study or carry out daily activities?

On how many days in the last three months did a headache spoil or prevent family, social, or leisure activities?

At follow-up telephonic contact (From telephonic consultation) T2

Frequency of headaches/month

(last three months)

Frequency of acute medication intake/Month

(last three months)

On how many days in the last three months did your headaches make it hard to study or carry out daily activities?

On how many days in the last three months did a headache spoil or prevent family, social, or leisure activities?

Do you have insight into the diagnosis given to you?                           Yes                           No             

(on follow-up, telephonic consultation)
